# A multicenter, randomized phase III trial of hetrombopag: a novel thrombopoietin receptor agonist for the treatment of immune thrombocytopenia

**DOI:** 10.1186/s13045-021-01047-9

**Published:** 2021-02-25

**Authors:** Heng Mei, Xiaofan Liu, Yan Li, Hu Zhou, Ying Feng, Guangxun Gao, Peng Cheng, Ruibin Huang, Linhua Yang, Jianda Hu, Ming Hou, Yazhou Yao, Li Liu, Yi Wang, Depei Wu, Liansheng Zhang, Changcheng Zheng, Xuliang Shen, Qi Hu, Jing Liu, Jie Jin, Jianmin Luo, Yun Zeng, Sujun Gao, Xiaohui Zhang, Xin Zhou, Qingzhi Shi, Ruixiang Xia, Xiaobao Xie, Zhongxing Jiang, Li Gao, Yuansong Bai, Yan Li, Junye Xiong, Runzi Li, Jianjun Zou, Ting Niu, Renchi Yang, Yu Hu

**Affiliations:** 1 Institute of Hematology, Union Hospital, Tongji Medical College, Huazhong University of Science and Technology, Wuhan, Hubei, 430022 China; 2 Thrombosis and Hemostasis Center, State Key Laboratory of Experimental Hematology, National Clinical Research Center for Hematological Disorders, Institute of Hematology and Blood Diseases Hospital, Chinese Academy of Medical Sciences and Peking Union Medical College, Tianjin Laboratory of Blood Disease Gene Therapy, CAMS Key Laboratory of Gene Therapy for Blood Diseases, Tianjin, 300020 China; 3 Department of Hematology, Institute of Hematology, West China Hospital, Sichuan University, Chengdu, 610041 Sichuan China; 4 Department of Hematology, Affiliated Cancer Hospital of Zhengzhou University, Zhengzhou, China; 5 Department of Hematopathology, The Second Affiliated Hospital of Guangzhou Medical University, Guangzhou, China; 6 The Blood Internal Medicine, The First Affiliated Hospital of Air Force Medical University, Xi’an, China; 7 Hematology Department, The First Affiliated Hospital of Guangxi Medical University, Nanning, China; 8 Hematology Department, The First Affiliated Hospital of Nanchang University, Nanchang, China; 9 Department of Hematology, The Second Hospital of Shanxi Medical University, Taiyuan, China; 10 Fujian Medical University Union Hospital, Fuzhou, China; 11 Department of Hematology, Qilu Hospital, Shandong University, Jinan, China; 12 Hematology Department, Baoji Central Hospital, Baoji, China; 13 Department of Hematopathology, The Second Affiliated Hospital of Air Force Medical University, Xi’an, China; 14 Department of Hematopathology, Shaanxi Provincial People’s Hospital, Xi’an, China; 15 Hematology Department, The First Affiliated Hospital of Soochow University, Suzhou, China; 16 Hematology Department, Lanzhou University Second Hospital, Lanzhou, China; 17 Hematology Department, The First Affiliated Hospital of USTC, Hefei, China; 18 Department of Hematology, Heping Hospital Affiliated To Changzhi Medical College, Changzhi, China; 19 Department of Hematology, Shanghai Municipal Hospital of Traditional Chinese Medicine, Shanghai, China; 20 The Third Xiangya Hospital of Central South University, Changsha, China; 21 Department of Hematology, The First Affiliated Hospital, Zhejiang University College of Medicine, Hangzhou, China; 22 Department of Hematology, The Second Hospital of Hebei Medical University, Shijiazhuang, China; 23 Department of Hematology, First Affiliated Hospital of Kunming Medical University, KunMing, China; 24 The First Hospital of Jilin University, Changchun, China; 25 Department of Hematology, Peking University People’s Hospital, Beijing, China; 26 Hematology Department, Wuxi People’s Hospital, Wuxi, China; 27 Hematology Department, The Second Affiliated Hospital of Nanchang University, Nanchang, China; 28 Hematology Department, The First Affiliated Hospital of Anhui Medical University, Hefei, China; 29 Hematology Department, The First People’s Hospital of Changzhou, Changzhou, China; 30 Department of Hematology, The First Affiliated Hospital of Zhengzhou University, Zhengzhou, China; 31 Department of Hematology, The Second Affiliated Hospital of Military Medical University PLA, Chongqing, China; 32 Hematology and Oncology, China–Japan Union Hospital of Jilin University, Changchun, China; 33 Hematology Department, The First Hospital of China Medical University, Shenyang, China; 34 Clinical Research & Development, Jiangsu Hengrui Medicine Co., Ltd, Shanghai, China

**Keywords:** Immune thrombocytopenia, Hetrombopag, Thrombopoietin receptor agonists, Platelet response

## Abstract

**Background:**

Hetrombopag, a novel thrombopoietin receptor agonist, has been found in phase I studies to increase platelet counts and reduce bleeding risks in adults with immune thrombocytopenia (ITP). This phase III study aimed to evaluate the efficacy and safety of hetrombopag in ITP patients.

**Methods:**

Patients who had not responded to or had relapsed after previous treatment were treated with an initial dosage of once-daily 2.5 or 5 mg hetrombopag (defined as the HETROM-2.5 or HETROM-5 group) or with matching placebo in a randomized, double-blind, 10-week treatment period. Patients who received placebo and completed 10 weeks of treatment switched to receive eltrombopag, and patients treated with hetrombopag in the double-blind period continued hetrombopag during the following open-label 14-week treatment. The primary endpoint was the proportion of responders (defined as those achieving a platelet count of ≥ 50 × 10^9^/L) after 8 weeks of treatment.

**Results:**

The primary endpoint was achieved by significantly more patients in the HETROM-2.5 (58.9%; odds ratio [OR] 25.97, 95% confidence interval [CI] 9.83–68.63; *p* < 0.0001) and HETROM-5 (64.3%; OR 32.81, 95% CI 12.39–86.87; *p* < 0.0001) group than in the Placebo group (5.9%). Hetrombopag was also superior to placebo in achieving a platelet response and in reducing the bleeding risk and use of rescue therapy throughout 8 weeks of treatment. The durable platelet response to hetrombopag was maintained throughout 24 weeks. The most common adverse events were upper respiratory tract infection (42.2%), urinary tract infection (17.1%), immune thrombocytopenic purpura (17.1%) and hematuria (15%) with 24-week hetrombopag treatment.

**Conclusions:**

In ITP patients, hetrombopag is efficacious and well tolerated with a manageable safety profile.

*Trial registration* Clinical trials.gov NCT03222843, registered July 19, 2017, retrospectively registered.

## Background

Primary immune thrombocytopenia (ITP) is characterized by increased platelet destruction and impaired platelet production, resulting in decreased platelet counts and increased bleeding risk [1]. *Bleeding* manifestations of primary ITP range from *skin* petechiae to life-threatening hemorrhages, such as gastrointestinal bleeding and intracerebral hemorrhage [2].

Typically, platelet counts of < 30 × 10^9^/L may be associated with an increased risk of spontaneous bleeding in adults with ITP [3]. The main principle of treatment strategies for ITP is to maintain a target platelet level of > 20–30 × 10^9^/L, at least for symptomatic patients, in order to prevent severe bleeding episodes since the risk of major bleeding increases below this level [4, 5] *. The standard f*irst-line therapy for chronic ITP is oral corticosteroids, administered to increase platelet counts. If patients do not respond to or have experienced recurrent relapse after first-line treatment, second-line therapy, such as thrombopoietin receptor agonists (TPO-RAs), rituximab or splenectomy, should be started [4, 6,7].

TPO-RAs have dramatically *changed the treatment landscape for ITP by providing patients with well*-*tolerated*, long-term *treatment* options. Romiplostim, eltrombopag and avatrombopag have been approved for chronic ITP adults in the US and European Union since 2008, 2009 and 2019, respectively. Eltrombopag was approved in 2018 in China and is the only TPO-RA for use in adults and children aged ≥ 12 years [7, 8]. Avatrombopag, the latest oral TPO-RA, was also confirmed to be superior to placebo with regard to a durable platelet response for chronic ITP in phase II and III trials [9, 10]. Nevertheless, the risk of bone marrow reticulin fiber formation with the subcutaneous administration of romiplostim, the risk of severe and potentially life-threatening hepatotoxicity with the use of eltrombopag, and blood clots caused by avatrombopag are inconvenient for ITP adults.

Hetrombopag olamine (hetrombopag) is the first small-molecule, nonpeptide oral TPO-RA developed in China. A preclinical study revealed that hetrombopag specifically enhanced the viability and promoted the growth of human thrombopoietin receptor-transfected murine 32D cells (32D-MPL) in hollow fibers implanted in nude mice, exhibiting much higher potency than eltrombopag in vivo [11]. A phase I clinical trial (NCT0240344) has evaluated the pharmacokinetics/pharmacodynamics and safety data of hetrombopag over 14 days in Chinese patients with chronic ITP (data submitted for publication). Another phase I clinical trial (NCT02614846) included a dose expansion design, and the preliminary results demonstrated that dose titration of hetrombopag (2.5–7.5 mg once daily) according to the patients’ dynamic platelet count was effective and generally well tolerated in ITP patients (data submitted for publication). However, because the sample sizes of the two clinical trials were limited and pharmacokinetics data after administration exhibited individual differences, initial dosages of once-daily 2.5 and 5 mg hetrombopag groups were considered simultaneously established to further explore the optimized choice of the initial dose and therapeutic dose in Chinese ITP patients. Hence, we conducted a randomized, multicenter, placebo-controlled phase III study to evaluate the efficacy and safety of hetrombopag in Chinese ITP patients who had not responded or had relapsed after previous treatment.

## Patients and methods

### Study design

This multicenter phase III study (NCT03222843) of hetrombopag in ITP adults, conducted at 33 sites in China (Additional file 1: Table S1), included a randomized, double-blind, placebo-controlled, 10-week treatment period, sequentially followed by an open-label 14-week treatment period, a less than six-week dose tapering to withdrawal period and an additional 24-week long-term extension period. During the 14-week open-label period, patients who received placebo and completed the 10-week double-blind treatment switched to receive eltrombopag. Herein, we report the results from the 10-week double-blind treatment period and the additional open-label 14-week treatment. This study was performed in accordance with the Declaration of Helsinki and the Good Clinical Practice guidelines. The protocol and all amendments were approved by the ethics committee at each site, and all patients provided written informed consent.

### Patients

Eligible patients were aged ≥ 18 years, diagnosed with primary ITP at least 6 months before randomization, had an insufficient response to or a relapse after splenectomy or at least one prior ITP drug (a platelet count of < 30 × 10^9^/L), and had a platelet count of < 30 × 10^9^/L within 48 h before the first dose of the study treatment. Maintenance immunosuppressive therapy for ITP (including corticosteroids, azathioprine, danazol, cyclosporin A, and mycophenolate mofetil) was allowed, provided that the dose was stable for at least 30 days before randomization. Previous rescue therapy (including methylprednisolone, platelet transfusion, or intravenous immunoglobulins [IVIG]) for ITP had to have been completed at least 2 weeks before randomization. Patients were not eligible if they were diagnosed with secondary thrombocytopenia or graded MF ≥ 2 myelofibrosis based on the European Consensus Scale [12] or thrombophilia or showed evidence of HIV, hepatitis C or B infections; other blood coagulation disorders; venous or arterial thrombosis; malignant tumors; liver cirrhosis; portal hypertension or congestive heart failure (New York Heart Association [NYHA] class III/IV); arrhythmia; myocardial infarction; atrial fibrillation; or corrected QT interval prolongation within the previous 3 months. Patients who had received other TPO-RA treatments or rituximab within 30 days before randomization or patients who did not respond to previous TPO-RAs (such as eltrombopag, romiplostim, etc.) were also excluded. Patients who had alanine aminotransferase or aspartate transaminase > 1.5 × upper limit of normal (ULN), total bilirubin or serum creatinine > 1.2 × ULN were also ineligible.

### Treatment interventions

In the double-blind treatment period, patients were randomly assigned to groups with an initial dosage of once-daily 2.5 or 5 mg hetrombopag (defined as the HETROM-2.5 group or HETROM-5 group) or to matching placebo groups at a ratio of 4:4:1:1 for 10 weeks or until withdrawal from the study. The active tablets and film-coated placebo were similar in color, shape, size and texture. Patients and all personnel involved in the trial conduct and interpretation (the investigators, investigational site personnel, and sponsor staff) were blinded to the treatment assignments (but not to the dose levels). Central randomization data were kept strictly confidential and were accessible until the time of unblinding. Key study withdrawal criteria included intolerable adverse events (AEs), protocol violations, and withdrawal by investigators.

During the open-label 14-week treatment period, patients treated with hetrombopag in the double-blind treatment period continued hetrombopag therapy with an initial dose that was administered at the end of week 10. Patients who had received placebo in the double-blind treatment period started eltrombopag treatment with an initial dosage of 25 mg once daily until week 24.

The dosage of hetrombopag/placebo should be initiated at a dosage of 2.5 mg or 5 mg once daily and can be titrated to a maximum of 7.5 mg once daily to maintain platelet counts between 50 × 10^9^/L and 250 × 10^9^/L. The dosage of eltrombopag should be initiated at a dosage of 25 mg once daily and can be adjusted to a maximum of 75 mg once daily to maintain platelet counts between 50 × 10^9^/L and 250 × 10^9^/L. The principles of dose adjustments for hetrombopag/placebo and eltrombopag in adult ITP are shown in Additional file 1: Table S2 and Table S3.

### Efficacy endpoints and assessments

Patients were assessed for platelet counts weekly in the double-blind treatment period and every 2 weeks in the open-label treatment period. Response to treatment was defined as a platelet count of ≥ 50 × 10^9^/L. The primary endpoint was the proportion of responders after 8 weeks of treatment. Prespecified subgroup analyses by baseline platelet count (< 10 × 10^9^/L or 10–30 × 10^9^/L), sex (male or female), age (18–65 or > 65 years), and prior splenectomy (yes or no) for the primary endpoint were also performed.

Other secondary efficacy endpoints included the proportions of responders after 3, 4, 5, 6, 7, and 8 weeks of treatment; within 8 weeks of treatment, the proportion of patients who responded at least once; the proportion of patients who responded at ≥ 75% of their assessments; the proportion of patients who achieved platelet counts ≥ 30 × 10^9^/L at least once that were at least twice their baseline platelet counts; the platelet counts at every scheduled visit; the time since the first dose to the first response; the *maximum continuous duration* and total duration of the response; the proportion of patients requiring protocol-defined rescue therapy (defined as either platelet transfusion or *IVIG* at the discretion of the investigators based on the clinical assessment), and the incidence and severity of bleeding symptoms; and within 24 weeks of treatment, the proportion of patients who responded at ≥ 75% of assessments, the *maximum continuous duration* of response, and the total duration of the response. Bleeding was assessed according to the World Health Organization (WHO) bleeding scale (grade 0, no bleeding; grade 1, petechiae; grade 2, mild blood loss; grade 3, gross blood loss; and grade 4, debilitating blood loss).

### Safety assessments

Continuous monitoring of AEs was performed. Clinical laboratory evaluations, physical examinations, electrocardiograms, ophthalmological examinations, and bone marrow biopsy were conducted and recorded at every scheduled study visit during the double-blind treatment period and open-label treatment period. Adverse events of special interest (AESIs) are potential drug-induced liver injuries. AEs were coded to the *preferred terms* of *the* Medical Dictionary for Regulatory Activities v22.0.

### Statistical analyses

The sample size calculation was based on the primary efficacy endpoint, with the assumption that 55% of subjects would respond to hetrombopag and 30% to placebo after 8 weeks of treatment. One hundred and thirty-two patients in each hetrombopag group and 66 in the placebo group guaranteed 90% power at a significance level of 5% (two-sided) to detect the difference between each hetrombopag group and placebo group, respectively, using Fisher’s exact test. Considering a dropout rate of 20%, 414 patients were randomized (165 patients for the HETROM-2.5 group, 165 for the HETROM-5 group, and 84 for the Placebo group).

All randomized patients who received at least one dose of the study treatment and had at least one assessment of their platelet count after randomization were included in the full analysis set. Patients who received at least one dose of the study treatment were included in the safety analysis set.

The primary endpoint in the HETROM-2.5 and HETROM-5 groups was compared with that in the placebo group using a logistic regression model adjusted for the baseline platelet count, with a noncompleters considered failure (NCF) imputation for patients who withdrew early from the study or patients with missing platelet count values at scheduled visits*.* The odds ratio (OR) and 95% confidence interval (CI) were provided. The baseline platelet count was defined as the last nonmissing value of the platelet count before the first treatment dose. The difference in the primary endpoint between the HETROM-5 group and placebo group was tested first; if and only if the test was statistically significant (i.e., 2-sided *p* ≤ 0.05), the difference between the HETROM-2.5 group and placebo group was tested.

A logistic regression model adjusted for the baseline platelet count was also conducted to compare the proportion of patients who responded at least once within 8 weeks, the proportion of patients who responded at ≥ 75% of assessments, and the proportion of patients who achieved platelet counts ≥ 30 × 10^9^/L at least once that were at least twice their baseline platelet count within 8 weeks using the NCF approach to impute missing values. The proportions of responders after 3, 4, 5, 6, 7, and 8 weeks of treatment were compared between the HETROM-2.5 or HETROM-5 group versus the Placebo group using a repeated measures model for binary data with time, treatment, and treatment-by-time interaction as fixed effects and baseline platelet count as a covariate. The generalized estimating equations method with the compound symmetry correlation structure was used to estimate the regression model parameters, and the corresponding OR and 95% CI values were calculated. The time from first dose to first response within 8 weeks was estimated using the Kaplan–Meier method, and patients without a platelet count of ≥ 50 × 10^9^/L within 8 weeks were deemed censored. The proportion of patients who required rescue therapy and the incidence of bleeding symptoms within 8 weeks were compared with Fisher’s exact test using the observed cases (OC) approach. The 95% CIs for the proportion of patients with a response in each group were computed by the *Clopper*–*Pearson* method if applicable. Data analyses were performed with SAS software (version 9.4).

## Results

### Patients

**Fig. 1:**
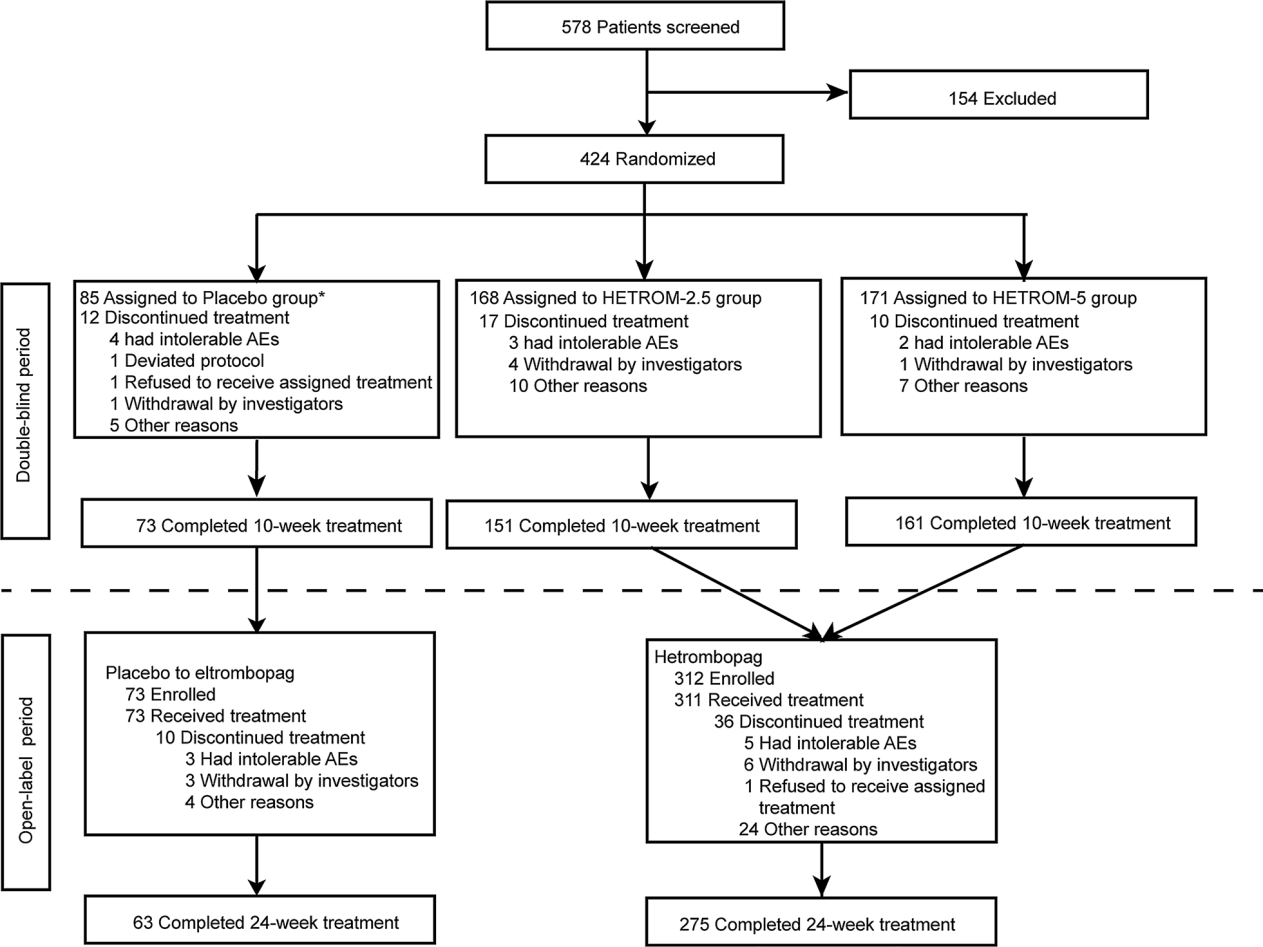
Trial profile. HETROM-2.5, the dose was titrated from an initial dosage of once-daily 2.5 mg hetrombopag; HETROM-5, the dose was titrated from an initial dosage of once-daily 5 mg hetrombopag; AEs, adverse events. *Eligible patients were randomly allocated at a ratio of 4:4:1:1 to the HETROM-2.5 group, the HETROM-5 group, and matching placebo groups. The two matching placebo groups were pooled as the Placebo group

Between June 30, 2017, and September 16, 2019, 578 patients were screened for eligibility, of whom 424 patients were eligible and randomized (4:4:1:1) into the HETROM-2.5 group (*n* = 168), the HETROM-5 group (*n* = 171), and the matching placebo groups (*n* = 43, 2.5 mg once daily as the initial dosage; *n* = 42, 5 mg once daily as the initial dosage), and the two placebo groups were pooled as the Placebo group (*n* = 85; Fig. 1). As of the data cutoff date on March 2, 2020, 151 patients (89.9%) in the HETROM-2.5 group, 161 (94.2%) in the HETROM-5 group, and 73 (85.9%) in the Placebo group completed the double-blind treatment period. The most frequent reason for treatment discontinuation from the double-blind period was intolerable AEs (*n* = 3, HETROM-2.5 group; *n* = 2, HETROM-5 group; *n* = 4, Placebo group). Of the 73 patients in the Placebo group who entered the open-label treatment phase, 63 patients (86.3%) completed the 14 weeks of treatment with eltrombopag. Of the 312 patients in the HETROM-2.5 and HETROM-5 groups who entered the open-label treatment phase, one patient (0.3%) did not continue treatment with hetrombopag due to a durable response. Of the 311 patients who continued hetrombopag treatment in the open-label treatment phase, 275 patients (88.4%) completed the preplanned 24-week treatment. The most frequent reasons for treatment discontinuation from the open-label treatment period were withdrawal by investigators (*n* = 6, hetrombopag group; *n* = 3, Placebo-Eltrombopag group) and intolerable AEs (*n* = 5, hetrombopag group; *n* = 3, Placebo-Eltrombopag group). The demographic and baseline clinical characteristics of the enrolled patients were similar between treatment groups (Table 1).

**Table 1:** Baseline characteristics

Characteristics	HETROM-2.5, *n* = 168	HETROM-5, *n* = 171	Placebo, *n* = 85
Age, median (range), years	38 (19–70)	41 (18–74)	42 (18–71)
Age, *n* (%)			
18–65 years	162 (96.4)	163 (95.3)	82 (96.5)
> 65 years	6 (3.6)	8 (4.7)	3 (3.5)
Height, median (range), cm	162.5 (147.0–182.0)	161.0 (147.0–182.0)	162.0 (150.0–185.5)
Weight, median (range), kg	61.0 (39.0–115.0)	62.5 (43.5–110.0)	63.0 (44.5–88.0)
BMI, median (range), kg/m^2^	23.4 (16.9–37.6)	24.4 (17.3–35.9)	23.6 (18.0–30.5)
Female, *n* (%)	122 (72.6)	119 (69.6)	60 (70.6)
Time since first ITP diagnosis, *n* (%)			
0.5–1 year	32 (19.0)	35 (20.5)	13 (15.3)
1–3 years	53 (31.5)	56 (32.7)	19 (22.4)
3–5 years	31 (18.5)	16 (9.4)	14 (16.5)
≥ 5 years	52 (31.0)	64 (37.4)	39 (45.9)
Prior splenectomy, *n* (%)	14 (8.3)	15 (8.8)	4 (4.7)
Bleeding (WHO bleeding scale grade 1–4), *n* (%)	108 (64.3)	93 (54.4)	52 (61.2)
Concomitant ITP medication at baseline, *n* (%)	168 (100.0)	168 (98.2)	83 (97.6)
Baseline platelet count, median (range), × 10^9^/L	13 (1–29)	13 (2–29)	13 (1–29)
Baseline platelet count, *n* (%)			
< 10 × 10^9^/L	64 (38.1)	58 (33.9)	35 (41.2)
10–30 × 10^9^/L	104 (61.9)	113 (66.1)	50 (58.8)

HETROM-2.5, dosage was titrated from an initial dose of once-daily 2.5 mg hetrombopag; HETROM-5, dosage was titrated from an initial dose of once-daily 5 mg hetrombopag; BMI, body mass index; ITP, immune thrombocytopenia; WHO, World Health Organization

### Primary endpoint

All 424 randomized patients were included in the full analysis set. Response to treatment (defined as platelet counts of ≥ 50 × 10^9^/L) after 8 weeks was achieved by significantly more patients in the HETROM-2.5 (58.9%) or HETROM-5 group (64.3%) than in the Placebo group (5.9%) (HETROM-2.5 versus Placebo: OR 25.97; 95% CI 9.83–68.63; HETROM-5 versus Placebo: OR 32.81; 95% CI 12.39–86.87; all *p* < 0.0001; Table 2). Subgroup analyses suggested generally consistent platelet count responses with patients treated with hetrombopag compared to placebo among subgroups, except patients with a baseline platelet count < 10 × 10^9^/L, elderly patients (> 65 years in age) or patients with prior splenectomy, due to the limited sample sizes (Additional file 1: Table S4).

**Table 2:** Primary endpoint and secondary efficacy endpoints within 8 weeks

Platelet response	HETROM-2.5, *n* = 168	HETROM-5, *n* = 171	Placebo, *n* = 85
Primary endpoint			
*n* (%)	99 (58.9)	110 (64.3)	5 (5.9)
OR (95% CI; *p*)^*,†^	25.97 (9.83–68.63; < 0.0001)	32.81 (12.39–86.87; < 0.0001)	–
Secondary efficacy end points within 8 weeks			
Proportion of patients who responded at least once			
*n* (%)	142 (84.5)	148 (86.5)	19 (22.4)
OR (95% CI; *p*)^*,†^	24.11 (11.76–49.40; < 0.0001)	27.83 (13.49–57.40; < 0.0001)	–
Proportion of patients who responded at ≥ 75% of assessments			
*n* (%)	55 (32.7)	98 (57.3)	2 (2.4)
OR (95% CI; *p*)^*,†^	24.63 (5.71–106.21; < 0.0001)	79.65 (18.36–345.53; < 0.0001)	–
Proportion of patients achieving platelet counts ≥ 30 × 10^9^/L at least once that were at least twice their baseline platelet counts			
*n* (%)	147 (87.5)	155 (90.6)	24 (28.2)
OR (95% CI; *p*)^*,†^	18.01 (9.31–34.85; < 0.0001)	24.93 (12.37–50.24, < 0.0001)	–
Maximum continuous duration of response			
n^#^	125	138	6
Median (range), days	22.0 (6.0–53.0)	22.0 (6.0–54.0)	8.5 (7.0–42.0)
Total duration of response			
n^#^	125	138	6
Median (range), days	23.0 (6.0–53.0)	33.0 (6.0–54.0)	8.5 (7.0–42.0)
Proportion of patients required rescue therapy			
n (%; 95% CI)^&^	22 (13.1; 8.4–19.2)	17 (9.9; 5.9–15.4)	32 (37.6; 27.4–48.8)
*p* value^¶^	< 0.0001	< 0.0001	–
Bleeding (WHO bleeding scale), *n* (%)^§^			
Yes	107 (64.1)	97 (56.7)	67 (78.8)
Grade 1	96 (57.5)	87 (50.9)	50 (58.8)
Grade 2	8 (4.8)	10 (5.8)	16 (18.8)
Grade 3	3 (1.8)	0	1 (1.2)
Grade 4	0	0	0
No	60 (35.9)	74 (43.3)	18 (21.2)
* p* value for present of bleed symptoms^¶^	0.0208	0.0005	–

Response to treatment was defined as a platelet count of ≥ 50 × 10^9^/L. HETROM-2.5, dosage was titrated from an initial dose of once-daily 2.5 mg hetrombopag; HETROM-5, dosage was titrated from an initial dose of once-daily 5 mg hetrombopag; OR, odd ratio; CI, confidence interval

^*^Hetrombopag versus Placebo

^†^Logistic regression analysis adjusted for baseline platelet count

^&^95% CI was calculated using the Clopper–Pearson method

^¶^*p* value from Fisher's exact test for comparison between Hetrombopag and Placebo

^§^HETROM-2.5 group, *n* = 167; one patient had no bleeding assessment

^#^Number of patients achieving response to treatment at consecutive scheduled visits

### Platelet count responses within 8 weeks since treatment

**Fig. 2:**
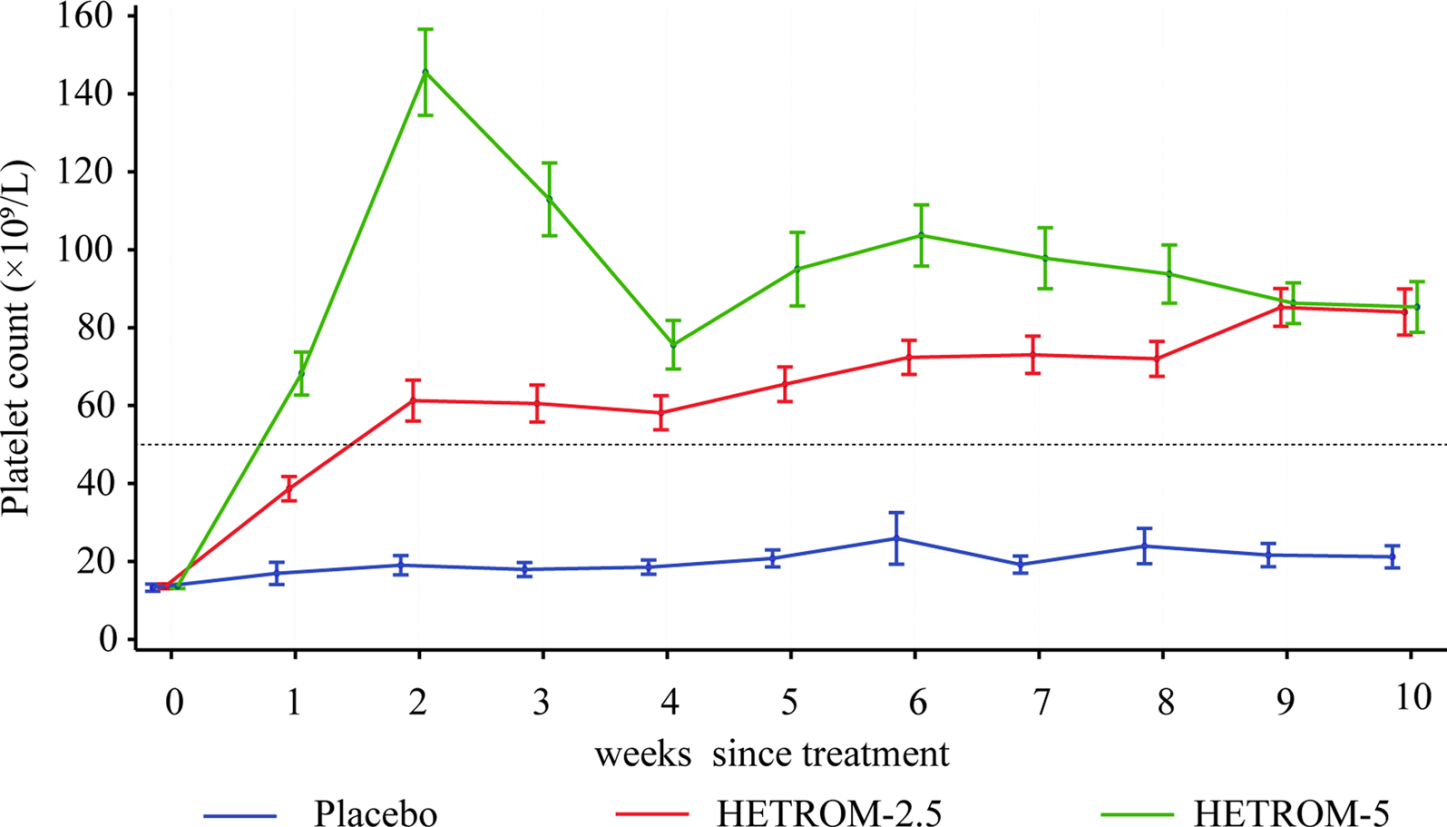
Platelet counts at every scheduled visit during the 10-week double-blind treatment period. HETROM-2.5, the dose was titrated from an initial dosage of once-daily 2.5 mg hetrombopag; HETROM-5, the dose was titrated from an initial dosage of once-daily 5 mg hetrombopag. The data are shown as means (standard errors)

Platelet counts at every scheduled assessment during the double-blind treatment period are indicated in Fig. 2. As presented in Additional file 1: Table S5, patients in the HETROM-2.5 or HETROM-5 group also had significantly greater odds of response after 3, 4, 5, 6, 7, and 8 weeks of treatment than those in the Placebo group (all *p* < 0.0001). A total of 142 patients (84.5%) in the HETROM-2.5 group and 148 patients (86.5%) in the HETROM-5 group responded to treatment at least once within 8 weeks, compared with 19 patients (22.4%) in the Placebo group (HETROM-2.5 versus placebo: OR 24.11; 95% CI 11.76–49.40; HETROM-5 versus placebo: OR 27.83; 95% CI 13.49–57.40; all *p* < 0.0001, Table 2). Throughout 8 weeks since treatment, treatment with hetrombopag at an initial dose of 2.5 or 5 mg was superior to placebo in terms of the proportion of patients who responded at ≥ 75% of the assessments (32.7%, HETROM-2.5 group; 57.3%, HETROM-5 group; 2.4%, Placebo group; HETROM-2.5 versus placebo: OR 24.63; 95% CI 5.71–106.21; HETROM-5 versus placebo: OR 79.65; 95% CI 18.36–345.53; all *p* < 0.0001, Table 2).

Furthermore, more patients in the HETROM-2.5 or HETROM-5 group than in the Placebo group achieved platelet counts ≥ 30 × 10^9^/L at least once that were at least twice their baseline platelet count within 8 weeks (87.5%, HETROM-2.5; 90.6%, HETROM-5; 28.2%, Placebo group; all *p* < 0.0001 compared with the Placebo group, Table 2).

**Fig. 3:**
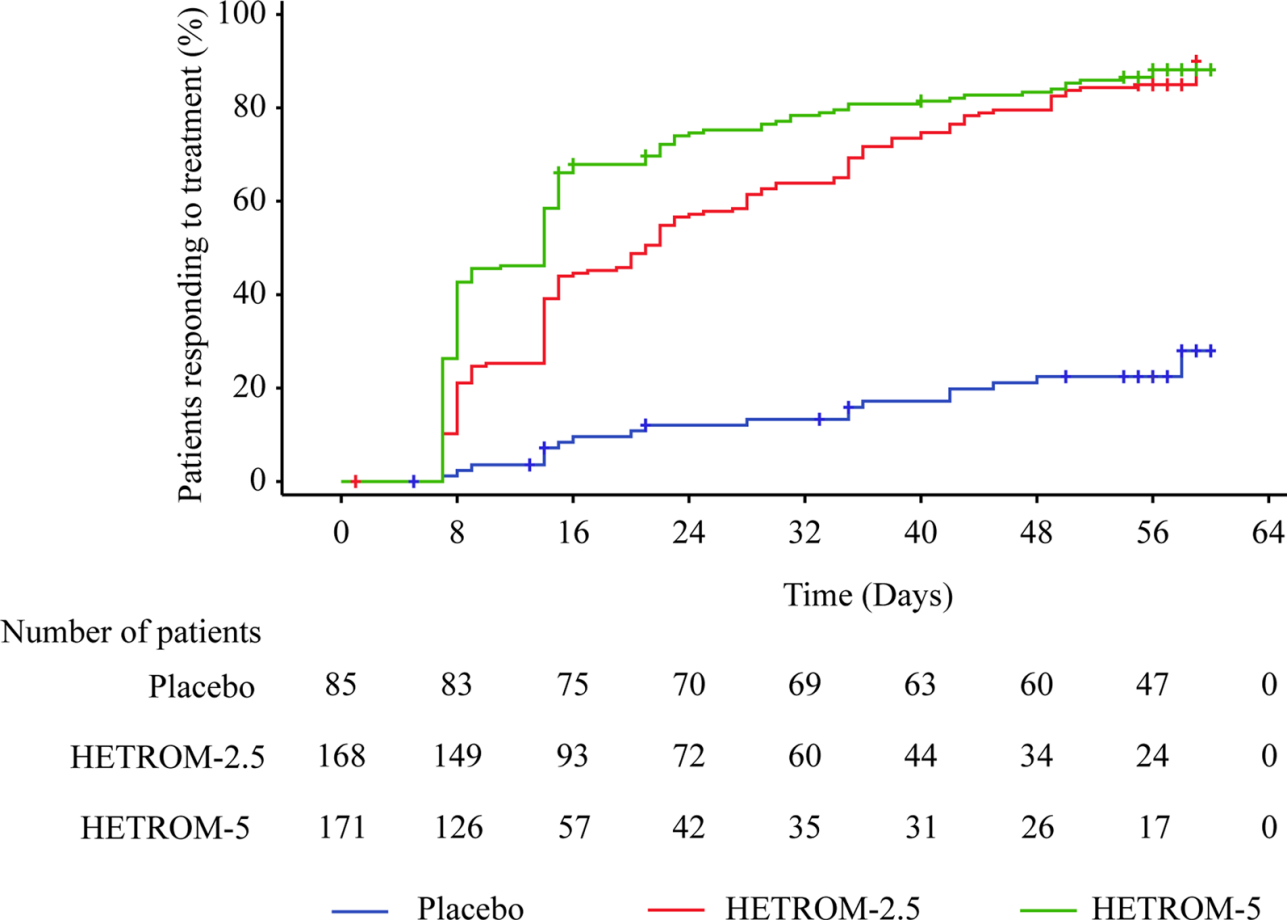
Proportions of patients achieving a first response (a platelet count of ≥ 50 × 10^9^/L) during the 8-week double-blind treatment period after the first dose of the study treatment. HETROM-2.5, the dose was titrated from an initial dosage of once-daily 2.5 mg hetrombopag; HETROM-5, the dose was titrated from an initial dosage of once-daily 5 mg hetrombopag

The median time from first dose to first response (platelet count ≥ 50 × 10^9^/L) within 8 weeks since treatment was 21.0 (95% CI 15.0–25.0) days and 14.0 (95% CI 8.0–14.0) days in the HETROM-2.5 and HETROM-5 groups, respectively (Fig. 3).

Among the 269 patients achieving a response to treatment at consecutive scheduled visits within 8 weeks (*n* = 125, HETROM-2.5 group; *n* = 138, HETROM-5 group; *n* = 6, Placebo group), the median maximum continuous durations of response were 22.0 (range 6.0–53.0) days, 22.0 (range 6.0–54.0) days, and 8.5 (range 7.0–42.0) days in the HETROM-2.5, HETROM-5, and Placebo group, respectively, and the median total durations of response were 23.0 (range 6.0–53.0) days, 33.0 (range 6.0–54.0) days, and 8.5 (range 7.0–42.0) days, respectively.

### Use of rescue therapy and presence of bleeding symptoms within 8 weeks since treatment

Twenty-two (13.1%) patients in the HETROM-2.5 group and 17 (9.9%) patients in the HETROM-5 group received protocol-defined rescue therapy within 8 weeks, and these percentages were significantly lower than that of the Placebo group (37.6%; all *p* < 0.0001, Table 2). As illustrated in Table 2, compared with the Placebo group (78.8%), significantly fewer patients in both the HETROM-2.5 group (64.1%, *p* = 0.0208) and HETROM-5 group (56.7%, *p* = 0.0005) had bleeding symptoms (WHO bleeding grades 1–4) within 8 weeks of treatment.

### Durable platelet count responses throughout 24 weeks of treatment with hetrombopag

Within 24 weeks of treatment, 39.9% (95% CI 32.4–47.7) of patients in the HETROM-2.5 group and 49.7% (95% CI 42.0–57.4) of patients in the HETROM-5 group responded at ≥ 75% of the assessments (Table 3). Among the 287 patients with hetrombopag who achieved a response to treatment at consecutive scheduled visits within 24 weeks of treatment with hetrombopag (*n* = 138, HETROM-2.5 group; *n* = 149, HETROM-5 group), the median maximum continuous durations of response were 64.0 (range 8.0–165.0) days and 64.0 (range 6.0–168.0) days, and the median total durations of response were 101.0 (8.0–165.0) days and 104.0 (6.0–168.0) days in the HETROM-2.5 and HETROM-5 groups, respectively. The results suggested that the durable platelet response could be maintained throughout 24 weeks of treatment in both the HETROM-2.5 and HETROM-5 groups (Table 3).

**Table 3:** Durable platelet counts response throughout 24-week treatment with hetrombopag

Endpoints with 24 weeks	HETROM-2.5, *n* = 168	HETROM-5, *n* = 171	Hetrombopag, *n* = 339
Proportion of patients who responded at ≥ 75% of their platelet count assessments			
n (%; 95% CI)^‡^	67 (39.9; 32.4–47.7)	85 (49.7; 42.0–57.4)	152 (44.8; 39.5–50.3)
Maximum continuous duration of response			
n^#^	138	149	287
Median (range), days	64.0 (8.0–165.0)	64.0 (6.0–168.0)	64.0 (6.0–168.0)
Total duration of response			
n^#^	138	149	287
Median (range), days	101.0 (8.0–165.0)	104.0 (6.0–168.0)	103.0 (6.0–168.0)

HETROM-2.5, dosage was titrated from an initial dose of once-daily 2.5 mg hetrombopag; HETROM-5, dosage was titrated from an initial dose of once-daily 5 mg hetrombopag

^‡^95% CI was calculated using the Clopper–Pearson method

^#^Number of patients achieving response to treatment at consecutive scheduled visits

### Treatment exposure

During the double-blind treatment period, the median duration of treatment exposure was 71.0 days (range, 2.0–76.0), 71.0 days (range, 16.0–74.0), and 70.0 days (range, 5.0–74.0) in the HETROM-2.5 group, HETROM-5 group, and Placebo group, respectively. During the open-label treatment period, the median duration of exposure to hetrombopag was 98.0 days (range, 6.0–121.0) in the hetrombopag group, and the median duration of eltrombopag exposure was 98.0 days (range, 20.0–106.0) in the Placebo-Eltrombopag group.

### Adverse events

The overall safety profile of hetrombopag during the double-blind and open-label treatment period is presented in Table 4. Within the 10-week double-blind treatment period, the overall incidence of AEs was comparable among the HETROM-2.5 group (91.7%), HETROM-5 group (94.7%) and Placebo group (95.3%). Of 424 patients, two patients (1.2%) in the HETROM-2.5 group, two patients (1.2%) in the HETROM-5 group, and four patients (4.7%) in the Placebo group experienced AEs leading to dose discontinuation, and six patients (3.6%) in the HETROM-2.5 group, 30 patients (17.5%) in the HETROM-5 group, and one patient (1.2%) in the Placebo group interrupted the study treatment or reduced the dose because of the occurrence of AEs.

**Table 4:** AEs within 10 weeks since treatment and within 24-week treatment

AE	Within 10 weeks since treatment			Within 24-week treatment	
	HETROM-2.5, *n* = 168	HETROM-5, *n* = 171	Placebo, *n* = 85	Hetrombopag, *n* = 339	Placebo-Eltrombopag, *n* = 85
Any AE, *n* (%)	154 (91.7)	162 (94.7)	81 (95.3)	332 (97.9)	85 (100.0)
AEs leading to dose discontinuation, *n* (%)	2 (1.2)	2 (1.2)	4 (4.7)	11 (3.2)	7 (8.2)
AEs leading to dose interruption or reduction, *n* (%)	6 (3.6)	30 (17.5)	1 (1.2)	47 (13.9)	7 (8.2)
AESI, *n* (%)	0	0	0	2 (0.6)	1 (1.2)
Most common AEs (≥ 10% of patients in either treatment group), *n* (%)					
Upper respiratory tract infection	44 (26.2)	45 (26.3)	29 (34.1)	143 (42.2)	39 (45.9)
Urinary tract infection	20 (11.9)	28 (16.4)	14 (16.5)	58 (17.1)	18 (21.2)
Platelet count increased	4 (2.4)	24 (14.0)	2 (2.4)	39 (11.5)	7 (8.2)
Blood urine present	24 (14.3)	21 (12.3)	11 (12.9)	49 (14.5)	13 (15.3)
Blood lactate dehydrogenase increased	12 (7.1)	21 (12.3)	6 (7.1)	40 (11.8)	9 (10.6)
Immune thrombocytopenic purpura	23 (13.7)	21 (12.3)	25 (29.4)	58 (17.1)	28 (32.9)
Red blood cells urine positive	24 (14.3)	14 (8.2)	13 (15.3)	47 (13.9)	17 (20.0)
Alanine aminotransferase increased	11 (6.5)	13 (7.6)	8 (9.4)	32 (9.4)	16 (18.8)
Diarrhea	17 (10.1)	13 (7.6)	1 (1.2)	39 (11.5)	3 (3.5)
Gingival bleeding	11 (6.5)	13 (7.6)	9 (10.6)	32 (9.4)	11 (12.9)
Headache	13 (7.7)	12 (7.0)	8 (9.4)	33 (9.7)	9 (10.6)
Hypokalemia	7 (4.2)	10 (5.8)	14 (16.5)	24 (7.1)	14 (16.5)
Protein urine present	11 (6.5)	7 (4.1)	8 (9.4)	19 (5.6)	9 (10.6)
Hepatic function abnormal	3 (1.8)	4 (2.3)	6 (7.1)	16 (4.7)	10 (11.8)
Any SAE, *n* (%)	16 (9.5)	15 (8.8)	17 (20.0)	49 (14.5)	21 (24.7)
Most common SAEs (≥ 2 patients in either treatment group), *n* (%)					
Thrombocytopenia*	15 (8.9)	9 (5.3)	16 (18.8)	34 (10.0)	18 (21.2)
Gastrointestinal hemorrhage	3 (1.8)	0	0	3 (0.9)	0
Cerebral hemorrhage	2 (1.2)	0	0	2 (0.6)	0
Death, *n* (%)	1 (0.6)	1 (0.6)	1 (1.2)	2 (0.6)	1 (1.2)

HETROM-2.5, dosage was titrated from an initial dose of once-daily 2.5 mg hetrombopag; HETROM-5, dosage was titrated from an initial dose of once-daily 5 mg hetrombopag; *thrombocytopenia was defined as platelet count was decreased compared with that observed at baseline. AE, adverse event; *S*AE, serious adverse event

The most common AEs were upper respiratory tract infection (26.2%), blood urine present (14.3%), red blood cells urine positive (14.3%), and immune thrombocytopenic purpura (13.7%) in the HETROM-2.5 group, upper respiratory tract infection (26.3%), urinary tract infection (16.3%), and increased platelet count (14.0%) in the HETROM-5 group, and upper respiratory tract infection (34.1%), immune thrombocytopenic purpura (29.4%), urinary tract infection (16.5%), and hypokalemia (16.5%) in the Placebo group. No AESI was reported in the double-blind treatment period.

Within 24 weeks of treatment, the most common AEs were upper respiratory tract infection (42.2%), urinary tract infection (17.1%), immune thrombocytopenic purpura (17.1%), and blood urine present (14.5%) in the hetrombopag group and upper respiratory tract infection (45.9%), immune thrombocytopenic purpura (32.9%), urinary tract infection (21.2%), and red blood cells urine positive (20.0%) in the Placebo-Eltrombopag group. One patient (1.2%) treated with eltrombopag and two patients (0.6%) treated with hetrombopag had AESIs during the open-label treatment period.

### Serious adverse events

Within 10 weeks after treatment, serious adverse events (SAEs) occurred in 17 patients (20.0%) in the Placebo group, which was higher than the incidences in the HETROM-2.5 group (9.5%) or HETROM-5 group (8.8%); however, the majority of patients had thrombocytopenia*, *as shown in Table 4. Five patients (3.0%) in the HETROM-2.5 group experienced hemorrhagic episodes, including gastrointestinal hemorrhage (3 patients, 1.8%) and cerebral hemorrhage (2 patients, 1.2%), but none of them was deemed treatment-related. In addition, one patient (0.6%) in the HETROM-5 group experienced an acute myocardial infarction (AMI) within two weeks after the first dose, which was associated with treatment-induced thrombocytosis; AMI was observed in neither the HETROM-2.5 group nor the Placebo group throughout the 24 weeks after treatment.

## Discussion

This is the first phase III trial to evaluate the efficacy and safety of hetrombopag in pure Chinese ITP patients who had not responded or had relapsed after previous treatment. Significant improvements were observed with hetrombopag treatment at an initial dosage of either 2.5 or 5 mg daily versus placebo for all primary and secondary efficacy endpoints during the double-blind treatment period.

The primary endpoint in this study was defined as the proportion of responders at week 8. We found that hetrombopag significantly increased the proportion (58.9%, HETROM-2.5 group; 64.3%, HETROM-5 group) of responders compared with that with placebo (5.9%, Placebo group) after 8 weeks of treatment (2.5 mg initial dose: OR 25.97 [95% CI 9.83–68.63]; 5 mg initial dose: 32.81 [95% CI 12.39–86.87]; all *p* < 0.0001). In several phase III clinical trials of eltrombopag in adults with chronic ITP, the primary endpoint was defined as the proportion of responders at week 6 and was achieved in 58.9% of patients in an international trial of once-daily 50 mg eltrombopag^14^, 60.0% in a Japanese population with once-daily 25 mg eltrombopag [14], and 57.7% in a Chinese population with once-daily 25 mg eltrombopag [15], compared with 0–16.2% in placebo-treated patients. In the present study, hetrombopag treatment promptly achieved a platelet response within 1–2 weeks. At Week 5 to Week 8, the proportion of responders was maintained at 57.7–59.5% with hetrombopag treatment at an initial dose of 2.5 mg and at 63.2–64.3% with hetrombopag treatment at an initial dose of 5 mg, which were comparable to the corresponding proportions for eltrombopag.

A sustained platelet response to hetrombopag at ≥ 75% of the platelet count assessments was found in 32.7% and 57.3% of patients with a 2.5 or 5 mg initial dose versus 2.4% of patients with placebo within 8 weeks after treatment, which was numerically higher than the 22.1% reported in a similar Chinese ITP population within 6 weeks after treatment with eltrombopag [15]. Although no formal hypothesis testing with a prespecified significance level for the direct comparison between the HETROM-2.5 and HETROM-5 groups was conducted in our phase III trial, as illustrated in Fig. 2, during the 10-week double-blind treatment period, an initial dosage of once-daily 2.5 hetrombopag could sustain the platelet level within a safe and stable range. Therapeutic effects between the HETROM-5 group and the HETROM-2.5 group were equivalent. Moreover, under the conditions that the proportions of patients achieving a platelet count response (a platelet count of ≥ 50 × 10^9^/L) were similar, compared with the HETROM-5 group, the fluctuations in platelet counts presented a more stable and smoother curve in the HETROM-2.5 group.

Our results also indicated that within 24 weeks after hetrombopag treatment, 44.8% (95% CI 39.5–50.3) of patients achieved a platelet response in at least 75% of their assessments. In addition, 287 out of 339 patients (84.6%) with hetrombopag achieved a response to treatment at consecutive scheduled visits within 24 weeks, and the median maximum continuous duration and median total duration of the platelet response were 64.0 (range, 6.0–168.0) days and 103.0 (range, 6.0–168.0) days, respectively. This phase III trial is still being continued to further explore the timing of hetrombopag withdrawal and long-term efficacy. Furthermore, the clinical benefit of ITP treatment is evaluated not only by the platelet response but also by bleeding symptoms and the use of rescue therapy. There were significantly lower incidences of bleeding symptoms (grade 1–4 based on the WHO bleeding scale) and use of rescue therapy with hetrombopag groups than placebo within 8 weeks since treatment.

In this phase III study, hetrombopag was shown to be well tolerated and to exhibit a safety profile that was comparable to placebo in ITP adults. Generally, treatment with hetrombopag at an initial dose of 2.5 or 5 mg was well tolerated, even though low rates of SAEs and study treatment discontinuations due to AEs were observed. Overall, the AE profile was comparable to that of placebo during the double-blind treatment period, except for slightly high incidences of increased platelet counts, diarrhea, and increased blood uric acid. The most frequent AEs were upper respiratory tract infection, urinary tract infection, immune thrombocytopenic purpura, blood urine present in patients on hetrombopag within 24 weeks of treatment.

Among the treatment-related AEs, the incidence of increased platelet counts was numerically higher in the HETROM-5 group (14%) than in the HETROM-2.5 group (2.4%) or the Placebo group (2.4%) within a 10-week double-blind treatment period. Of these, 23 patients (13.5%) in the HETROM-5 group, 3 patients (1.8%) in the HETROM-2.5 group and 1 patient (1.2%) in the Placebo group were considered as to have treatment-related AEs at the *investigator's* discretion. Additionally, during the double-blind treatment period, AEs leading to dose interruption or reduction occurred in 17.5% of patients in the HETROM-5 group, which was higher than the 3.6% in the HETROM-2.5 group and the 1.2% in the placebo group, and most of these patients had increased platelet counts (13.5%; HETROM-5 group). One patient in the HETROM-5 group had an AMI during the double-blind treatment period due to treatment-induced thrombocytosis; this patient recovered after hospitalization and resumed the study treatment. These findings support that an initial dosage of once-daily 2.5 mg hetrombopag can reduce AEs induced by increased platelet counts, thereby decreasing the occurrence of potential thromboembolic events and gradually achieving the optimal clinical therapeutic dose through dose adjustments based on measuring the platelet count at every scheduled visit. Therefore, the usage of once-daily 2.5 mg hetrombopag as the initial dose is recommended for Chinese ITP patients. During the open-label treatment period, the AE profile of patients switched to eltrombopag was in line with those reported in previous trials [13–17]. The incidences of thromboembolic events, hepatotoxicity, cataracts, prolonged QT interval, and myelofibrosis in patients treated with other approved TPO-RAs are worth mentioning, with such drugs including romiplostim, eltrombopag and avatrombopag [10, 15, 18]. Although not all of these conditions were prespecified as AESIs in our phase III trial, these mentioned treatment-related AEs also occurred in patients receiving 24-week hetrombopag (*n* = 2, thromboembolic events, including one patient AMI and another with s*ubclavian vein thrombosis*; *n* = 27, increased alanine aminotransferase; *n* = 24, increased aspartate aminotransferase; *n* = 1, cataracts; *n* = 1, prolonged QT interval with QTc 469 ms; *n* = 2, graded MF 1/2 myelofibrosis), who were all cured or recovered after active treatments. However, the AE spectra of various TPO-RAs do not overlap. Specific AEs, for example, transaminitis and cataracts, are more commonly observed in patients with eltrombopag; pain after administration (extremity pain, abdominal pain or shoulder pain) is more prone to occur in patients with romiplostim, which might be attributed to the development of neutralizing antibodies; and headache is the most frequent AE in patients with avatrombopag [8]. A previous study also demonstrated that patients who were intolerant of romiplostim could successfully switch to eltrombopag [19], and the absence of overlapping AEs encouraged switching when TPO-RA was discontinued because the AE was not due to class effects [20].

The majority of ITP cases are caused by autoantibodies generated against platelets, which also affect platelet clearance and the efficacy of first-line therapies through different mechanisms [21–25]. Additionally, there are reports that cytotoxic T lymphocytes (CTLs) contribute to ITP [26,27]. We did not check TPO and megakaryocyte (MK) levels or autoantibodies and *CTLs* against platelets before and after hetrombopag treatment. A previous study observed that eltrombopag favored human MK differentiation and platelet production in a dose-dependent manner [28]. Further studies are required to explore whether the regulatory effect of hetrombopag on MKs is in line with eltrombopag. The prevailing theory posits that circulating TPO levels are maintained through its clearance by platelets and MKs via surface c-Mpl receptor internalization, and whether increased platelets could cause increased clearance of TPO and subsequently lead to decreased TPO levels is still uncertain. In addition, Xu M et al. demonstrated that *glycoprotein Iba* (*GPIba*) is a prerequisite for hepatic TPO generation [29]. Moreover, a recent study in a mouse model suggested that TPO-RAs inhibited the production of antiplatelet antibodies, but how this occurred was still unknown [30]. Previous studies have found that TPO-RA treatment might play a role in improving regulatory T cell function to restore immune tolerance and could shift the balance of *Fcγ receptors* toward the inhibitory receptor IIb on monocytes in ITP patients [31, 32], indicating that TPO-RA might function in the processes of immune regulation in addition to increasing MK proliferation. However, there are still no studies about the regulation of GPIba by TPO receptor agonists and the effect of hetrombopag on CTL levels, which are both interesting investigative directions for the near future.

This study has several limitations. First, some TPO-RAs have already been made available and are widely employed for ITP in clinical practice, and our trial did not use a previously approved TPO-RA as a control because no TPO-RA was approved in China during the study design. Currently, *eltrombopag* is the *only* TPO-RA *approved in China, and the relative efficacy and safety of hetrombopag compared with eltrombopag warrant further exploration.* Second, this study reported the efficacy and safety of *hetrombopag with* a 24-week treatment duration, and the efficacy and safety of dose tapering to withdrawal and long-term treatment with hetrombopag are still ongoing.

## Conclusions

This phase III trial was the first to demonstrate that hetrombopag was superior to placebo in increasing platelet counts and reducing bleeding risk and the use of rescue therapy over the 8-week treatment period in ITP patients with an insufficient response or relapse after prior ITP treatment or splenectomy; the platelet response was maintained with extended exposure to hetrombopag throughout the 24-week treatment period. Hetrombopag was generally well tolerated, with a manageable safety profile. An initial dosage of 2.5 mg hetrombopag once daily might be appropriate for Chinese patients with ITP.

## Supplementary Information

**Additional file 1: Table S1.** Investigators per site. **Table S2.** Dose adjustments of hetrombopag during the double-blind treatment period and open-label treatment period. **Table S3.** Dose adjustments of eltrombopag for patients in the Placebo-eltrombopag group during the double-blind treatment period and open-label treatment period. **Table S4.** Platelet responses (platelet counts ≥ 50 × 10^9^/L at week 8) were observed across all subgroups of baseline platelet counts, sex, age, and splenectomy status. **Table S5.** Proportion of responders at different time points within 8 weeks after treatment.

## Data Availability

The datasets used and/or analyzed during the current study are available from the corresponding author on reasonable request.

## Abbreviations

ITP: Immune thrombocytopenia
IVIG: Intravenous immunoglobulins
TPO-RA: Thrombopoietin receptor agonist
NYHA: New York Heart Association
ULN: Upper limit of normal
AE: Adverse event
WHO: World Health Organization
AESI: Adverse event of special interest
NCF: Noncompleters considered failure
OR: Odds ratio
CI: Confidence interval
OC: Observed cases
SAE: Serious adverse event
AMI: Acute myocardial infarction
MK: Megakaryocyte
CTL: Cytotoxic T lymphocyte
GPIba: Glycoprotein Iba
