# Supplementary material for: A multicenter, randomized phase III trial of hetrombopag: a novel thrombopoietin receptor agonist for the treatment of immune thrombocytopenia
Source: J Hematol Oncol. 2021 Feb 25;14:37. doi: 10.1186/s13045-021-01047-9 (PMC7905908; doi:10.1186/s13045-021-01047-9)
Supplement: Supplementary file 1 — Additional file 1: Table S1. Investigators per site. Table S2. Dose adjustments of hetrombopag during the double-blind treatment period and open-label treatment period. Table S3. Dose adjustments of eltrombopag for patients in the Placebo-eltrombopag group during the double-blind treatment period and open-label treatment period. Table S4. Platelet responses (platelet counts ≥ 50 × 109/L at week 8) were observed across all subgroups of baseline platelet counts, sex, age, and splenectomy status. Table S5. Proportion of responders at different time points within 8 weeks after treatment. [file 13045_2021_1047_MOESM1_ESM.docx]

# Appendix Tables

# Additional file 1: Table S1. Investigators per site.

| Study site | Principal Investigator | Number of patients enrolled |
| --- | --- | --- |
| State Key Laboratory of Experimental Hematology, National Clinical Research Center for Hematological Disorders, Institute of Hematology and Blood Diseases Hospital, Chinese Academy of Medical Sciences and Peking Union Medical College, Tianjin Laboratory of Blood Disease Gene Therapy, CAMS Key Laboratory of Gene Therapy for Blood Diseases | Renchi yang | 51 |
| Union Hospital, Tongji Medical College, Huazhong University of Science and Technology | Yu Hu | 49 |
| Affiliated Cancer Hospital of Zhengzhou University | Hu Zhou | 34 |
| The Second Affiliated Hospital of Guangzhou Medical University | Ying Feng | 28 |
| The First Affiliated Hospital of Air Force Medical University | Guangxun Gao | 22 |
| West China Hospital, Sichuan University | Ting Niu | 19 |
| The First Affiliated Hospital of Guangxi Medical University | Peng Cheng | 19 |
| The First Affiliated Hospital of Nanchang University | Ruibin Huang | 17 |
| The Second Hospital of Shanxi Medical University | Linhua Yang | 17 |
| Fujian Medical University Union Hospital | Jianda Hu | 17 |
| Department of Hematology, Qilu Hospital, Shandong University | Ming Hou | 14 |
| Baoji Central Hospital | Yazhou Yao | 13 |
| The Second Affiliated Hospital of Air Force Medical University | Li Liu | 12 |
| Shaanxi Provincial People's Hospital | Yi Wang | 12 |
| The First Affiliated Hospital of Soochow University | Depei Wu | 11 |
| Lanzhou University Second Hospital | Liansheng Zhang | 10 |
| The First Affiliated Hospital of USTC | Changcheng Zheng | 9 |
| Heping Hospital Affiliated to Changzhi Medical College | Xuliang Shen | 9 |
| Shanghai Municipal Hospital of Traditional Chinese Medicine | Qi Hu | 9 |
| The Third Xiangya Hospital of Central South University | Jing Liu | 7 |
| The First Affiliated Hospital, Zhejiang University College of Medicine | Jie Jin | 6 |
| The Second Hospital of Hebei Medical University | Jianmin Luo | 5 |
| First Affiliated Hospital of Kunming Medical University | Yun Zeng | 5 |
| The First Hospital of Jilin University | Sujun Gao | 5 |
| Peking University People's Hospital | Xiaohui Zhang | 4 |
| Wuxi People's Hospital | Xin Zhou | 4 |
| The Second Affiliated Hospital of Nanchang University | Qingzhi Shi | 3 |
| The First Affiliated Hospital of Anhui Medical University | Ruixiang Xia | 3 |
| The First People's Hospital of Changzhou | Xiaobao Xie | 3 |
| The First Affiliated Hospital of Zhengzhou University | Zhongxing Jiang | 3 |
| The Second Affiliated Hospital of Military Medical University PLA | Li Gao | 2 |
| China–Japan Union Hospital of Jilin University | Yuansong Bai | 1 |
| The First Hospital of China Medical University | Yan Li | 1 |

# Additional file 1: Table S2. Dose adjustments of hetrombopag during the double-blind treatment period and open-label treatment period.

|  | Current dosage | Platelet count | | | |
| --- | --- | --- | --- | --- | --- |
|  |  | <50×10^9^/L | ≥50-150×10^9^/L | >150-250 ×10^9^/L | ≥250×10^9^/L |
| Week 0-Week 2 since treatment | 2.5 mg once daily | Use a fixed dosage of 2.5 mg once daily to maintain platelet counts, and the dose could be increased based on safety if necessary. | Maintenance dosage of 2.5 mg once daily | Decrease the dosage to 2.5 mg once every 2 days. | Stop hetrombopag until the platelet count is ≤ 100×10^9^/L; reinitiate therapy at a dosage of 2.5 mg administered every 2 days. |
|  | 5 mg once daily | Use a fixed dosage of 5 mg once daily to maintain platelet counts, and the dose could be increased based on safety if necessary. | Maintenance dosage of 5 mg once daily | Decrease the dosage to 3.75 mg once daily. | Stop hetrombopag until the platelet count is ≤ 100×10^9^/L; reinitiate therapy at a dosage of 3.75 mg administered once daily. |
| Week 3-Week 14 since treatment | 2.5 mg once daily | Increase the dosage to 3.75 mg once daily | Maintenance dosage of 2.5 mg once daily | Decrease the dosage to 2.5 mg once every 2 days. | Stop hetrombopag until the platelet count is ≤ 100×10^9^/L; reinitiate therapy at a dosage of 2.5 mg administered once daily. |
|  | 3.75 mg once daily | Increase the dosage to 5 mg once daily | Maintenance dosage of 3.75 mg once daily | Decrease the dosage to 2.5 mg once daily. | Stop hetrombopag until the platelet count is ≤ 100×10^9^/L; reinitiate therapy at a dosage of 2.5 mg administered once daily. |
|  | 5 mg once daily | Increase the dosage to 7.5 mg once daily | Maintenance dosage to 5 mg once daily | Decrease the dosage to 3.75 mg once daily. | Stop hetrombopag until the platelet count is ≤ 100×10^9^/L; reinitiate therapy at a dosage of 3.75 mg administered once daily. |
|  | 7.5 mg once daily | Maintenance dosage of 7.5 mg once daily | Maintenance dosage of 7.5 mg once daily | Decrease the dosage to 5 mg once daily. | Stop hetrombopag until the platelet count is ≤ 100×10^9^/L; reinitiate therapy at a dosage of 5 mg administered once daily. |

# Additional file 1: Table S3. Dose adjustments of eltrombopag for patients in the placebo-eltrombopag group during the double-blind treatment period and open-label treatment period.

| Current dosage | Platelet count | | | |
| --- | --- | --- | --- | --- |
|  | <50×10^9^/L | ≥50-150×10^9^/L | >150-250 ×10^9^/L | ≥250×10^9^/L |
| 25 mg every 2 days | Increase the dosage to 25 mg once daily | Maintenance dosage of 25 mg once every 2 days | Decrease the dosage to 25 mg once every few days | Stop eltrombopag until the platelet count is ≤100×10^9^/L; reinitiate therapy at a dosage of 25 mg administered once every few days. |
| 25 mg once daily | Increase the dosage to 50 mg once daily | Maintenance dosage of 25 mg once daily | Decrease the dosage to 25 mg once every 2 days. | Stop eltrombopag until the platelet count is ≤100×10^9^/L; reinitiate therapy at a dosage of 25 mg administered once every 2 days. |
| 50 mg once daily | Increase the dosage to 75 mg once daily | Maintenance dosage of 50 mg once daily | Decrease the dosage to 25 mg once daily. | Stop eltrombopag until the platelet count is ≤100×10^9^/L; reinitiate therapy at a dosage of 25 mg administered once daily. |
| 75 mg once daily | Maintenance dosage of 75 mg once daily | Maintenance dosage of 75 mg once daily | Decrease the dosage to 50 mg once daily. | Stop eltrombopag until the platelet count is ≤100×10^9^/L; reinitiate therapy at a dosage of 50 mg administered once daily. |

# Additional file 1: Table S4. Platelet responses (platelet counts ≥50$\boldsymbol{\times}$10^9^/L at week 8) were observed across all subgroups of the baseline platelet count, sex, age, and splenectomy status. HETROM-2.5, the dose was titrated from an initial dosage of once-daily 2.5 mg hetrombopag; HETROM-5, the dose was titrated from an initial dosage of once-daily 5 mg hetrombopag; OR, odds ratio; CI, confidence interval. ^*^Hetrombopag versus Placebo. ^†^Logistic regression analysis adjusted for the baseline platelet count.

| Variables | Subgroups | n (%) | OR (95% CI; p) * † |
| --- | --- | --- | --- |
| Baseline platelet count | | | |
| <10×10^9^/L | HETROM-2.5 (n=64) | 28 (43.8) | Not calculated |
|  | HETROM-5 (n=58) | 30 (51.7) | Not calculated |
|  | Placebo (n=35) | 0 | .. |
| 10-30×10^9^/L | HETROM-2.5 (n=104) | 71 (68.3) | 19.55 (7.10-53.86; <0.0001) |
|  | HETROM-5 (n=113) | 80 (70.8) | 22.37 (8.11-61.70; <0.0001) |
|  | Placebo (n=50) | 5 (10.0) | .. |
| Gender | | | |
| Male | HETROM-2.5 (n=46) | 27 (58.7) | 39.24 (4.74-324.97; 0.0007) |
|  | HETROM-5 (n=52) | 30 (57.7) | 41.11 (4.96-340.85; 0.0006) |
|  | Placebo (n=25) | 1 (4.0) | .. |
| Female | HETROM-2.5 (n=122) | 72 (59.0) | 22.48 (7.51-67.32; <0.0001) |
|  | HETROM-5 (n=119) | 80 (67.2) | 30.84 (10.25-92.81; <0.0001) |
|  | Placebo (n=60) | 4 (6.7) | .. |
| Age | | | |
| 18-65 years | HETROM-2.5 (n=162) | 95 (58.6) | 31.39 (10.78-91.46; <0.0001) |
|  | HETROM-5 (n=163) | 102 (62.6) | 37.47 (12.84-109.36; <0.0001) |
|  | Placebo (n=82) | 4 (4.9) | .. |
| >65 years | HETROM-2.5 (n=6) | 4 (66.7) | Not calculated |
|  | HETROM-5 (n=8) | 8 (100.0) | Not calculated |
|  | Placebo (n=3) | 1 (33.3) | .. |
| Prior splenectomy | | | |
| Yes | HETROM-2.5 (n=14) | 4 (28.6) | Not calculated |
|  | HETROM-5 (n=15) | 8 (53.3) | Not calculated |
|  | Placebo (n=4) | 0 | .. |
| No | HETROM-2.5 (n=154) | 95 (61.7) | 27.65 (10.39-73.59; <0.0001) |
|  | HETROM-5 (n=156) | 102 (65.4) | 32.30 (12.12-86.06; <0.0001) |
|  | Placebo (n=81) | 5 (6.2) | .. |

# **Additional file 1: Table S5. Proportion of responders at different time points within 8 weeks after treatment.** Response to treatment was defined as a platelet count of ≥50×10^9^/L. HETROM-2.5, the dose was titrated from an initial dosage of once-daily 2.5 mg hetrombopag; HETROM-5, the dose was titrated from an initial dosage of once-daily 5 mg hetrombopag; OR, odds ratio; CI, confidence interval. *The proportions of responders after 3, 4, 5, 6, 7, and 8 weeks of treatment were compared between the HETROM-2.5 or HETROM-5 group versus the Placebo group using a repeated measures model for binary data with time, treatment, and treatment-by-time interaction as fixed effects and baseline platelet count as a covariate. ^‡^ The generalized estimating equations method with the compound symmetry correlation structure was used to estimate the regression model parameters, and corresponding OR and 95% CI values were calculated.

| Platelet response | | HETROM-2.5, n=168 | HETROM-5, n=171 | Placebo, n=85 |
| --- | --- | --- | --- | --- |
| Secondary efficacy endpoints | | | | |
| 3 weeks since treatment | n (%) | 71 (42.3) | 104 (60.8) | 4 (4.7) |
|  | OR (95% CI; p) ^* ‡^ | 16.99 (5.75-50.25; <0.0001) | 37.31 (12.62-110.31; <0.0001) | .. |
| 4 weeks since treatment | n (%) | 71 (42.3) | 93 (54.4) | 5 (5.9) |
|  | OR (95% CI; p) ^* ‡^ | 13.40 (5.01-35.88; <0.0001) | 22.17 (8.27-59.39; <0.0001) |  |
| 5 weeks since treatment | n (%) | 97 (57.7) | 109 (63.7) | 7 (8.2) |
|  | OR (95% CI; p) ^* ‡^ | 18.27 (7.68-43.45; <0.0001) | 23.32 (9.84-55.22; <0.0001) | .. |
| 6 weeks since treatment | n (%) | 99 (58.9) | 110 (64.3) | 6 (7.1) |
|  | OR (95% CI; p) ^* ‡^ | 22.82 (9.06-57.49; <0.0001) | 28.38 (11.29-71.33; <0.0001) | .. |
| 7 weeks since treatment | n (%) | 100 (59.5) | 108 (63.2) | 5 (5.9) |
|  | OR (95% CI; p) ^* ‡^ | 28.55 (10.51-77.54; <0.0001) | 32.74 (12.14-88.26; <0.0001) | .. |
| 8 weeks since treatment | n (%) | 99 (58.9) | 110 (64.3) | 5 (5.9) |
|  | OR (95% CI; p) ^* ‡^ | 27.79 (10.36-74.54; <0.0001) | 34.56 (12.90-92.60; <0.0001) | .. |
